# Knockdown of TRIM65 inhibits autophagy and cisplatin resistance in A549/DDP cells by regulating miR-138-5p/ATG7

**DOI:** 10.1038/s41419-019-1660-8

**Published:** 2019-06-03

**Authors:** Xufeng Pan, Yong Chen, Yuzhou Shen, Jicheng Tantai

**Affiliations:** Department of Thoracic Surgery, Shanghai Chest Hospital, Shanghai Jiao Tong University, Shanghai, China

**Keywords:** Lung cancer, Cell biology

## Abstract

Cisplatin resistance is the main cause of treatment failure in patients with non-small-cell lung cancer (NSCLC). Autophagy is a key mechanism of resistance to chemotherapy. Given that tripartite motif (TRIM)-containing proteins are involved in the regulation of autophagy and chemoresistance, we aimed to study the functions of TRIM protein members in autophagy-mediated chemoresistance of NSCLC. We found that TRIM65 was significantly increased in cisplatin-resistant NSCLC cell line (A549/DDP) as compared to the parental cell line (A549). Knockdown of TRIM65 can enhance cisplatin-induced apoptosis and inhibit autophagy in A549/DDP cells, as indicated by Annexin V/PI staining, caspase3 activity test, and LC3-II immunofluorescence staining. Additionally, knockdown of TRIM65 significantly decreased the expression of an important autophagy mediator, ATG7, which was a potential target of miR-138-5p. miR-138-5p inhibitor significantly abolished the effects of TRIM65 knockdown on autophagy and cisplatin-induced apoptosis. Moreover, TRIM65 induced the ubiquitination and degradation of TNRC6A, resulting in the suppressed expression of miR-138-5p. TRIM65 knockdown inhibited the growth of tumors derived from A549/DDP cells. Furthermore, cisplatin-resistant NSCLC tissues displayed higher expression of TRIM65 mRNA and lower expression of miR-138-5p as compared to cisplatin non-resistant ones. miR-138-5p expression was negatively correlated with TRIM65 mRNA in NSCLC tissues. Collectively, the present study indicates that TRIM65 knockdown attenuates autophagy and cisplatin resistance in A549/DDP cells via regulating miR-138-5p.

## Introduction

Lung cancer ranks the first among all cancer-related deaths worldwide, and more than 1 million people die from lung cancer annually^1^. Approximately 80% of lung cancer cases are non-small-cell lung cancer (NSCLC)^2^. Surgery is the main approach to treat early-stage NSCLC. However, most of the patients have little opportunity to receive surgery because they have locally advanced disease or distant metastasis at diagnosis^3^. For these patients, the platinum-based drug, cisplatin, is currently the standard drug treatment. Unfortunately, acquired resistance causes treatment failure and the extremely poor prognosis of these individuals^4,5^. Therefore, a deeper understanding of the chemoresistance mechanisms may help to develop strategies for overcoming drug resistance and improving clinical outcomes^6^.

Autophagy, a evolutionally conserved process, is genetically regulated by a family of autophagy-related genes (ATGs), and allows the orderly degradation and recycling of intracellular organelles and cytoplasmic proteins^7,8^. Recently, autophagy has been identified as an important mechanism of resistance to chemotherapy^9,10^, and manupulation of autophagy has emerged as a promising strategy to overcome chemoresistance in cancer therapy^11^. For instance, hydroxychloroquine (CQ), an autophagy inhibitor, has been used to enhance the sensitivity to chemotherapy in NSCLC patients^12^. MicroRNAs (miRNAs), a class of small endogenous non-coding RNAs, can regulate gene transcription by binding to the 3′-untranslated region (3′-UTR) of target mRNAs^13^. Increasing evidence has indicated that miRNAs may play a role in chemoresistance in some cancer cells. For example, upregulation of miR-21–3p increases resistance to cisplatin by targeting NAV3 in ovarian cancer cells^14^. Overexpression of miR-15b and miR-16 reduces the resistance to vincristine by targeting Bcl-2 in gastric cancer cells^15^. miR-326 prevents multidrug resistance in breast cancer cells by targeting multidrug resistance-associated protein (MRP-1)^16^. By a miRNA array, Wang et al. has identified 14 significantly differentiated expressed miRNAs in cisplatin-resistant NSCLC cell line (A549/DDP) as compared to the parental cell line (A549)^17^. More interesting, several miRNAs have been found to enhance chemosensitivity and apoptosis of cancer cells by inhibiting autophagy, e.g. miR-21^18^, miR-26,^19^ and miR-101^20^.

Tripartite motif (TRIM)-containing proteins have more than 80 members in humans, most of which could be defined as E3 ubiquitin ligase^21^. TRIM proteins are involved in the regulation of development^21^, immunity^22^, carcionogenesis^23^, autophagy^24^ and chemoresistance^25–30^. It has been rarely explored whether TRIM proteins affect the chemoresistance and autophagy in NSCLC cells. In the current study, we found that TRIM65 was significantly increased in A549/DDP cells as compared to A549 cells. Knockdown of TRIM65 can inhibit autophagy and enhance cisplatin-induced apoptosis in A549/DDP cells. As TRIM65 is reported as a negative regulator of miRNA activity by forming stable complexes with trinucleotide repeat containing six (TNRC6) proteins^31^, we further explored the possible downstream miRNAs, which could target the autophagy mediator, ATG7. Our study may provide new insight into the role of TRIM65 in the autophagy-mediated chemoresistance of NSCLC.

## Results

### TRIM65 expression was upregulated in A549/DDP cells and involved in cisplatin-induced apoptosis

**Fig. 1:**
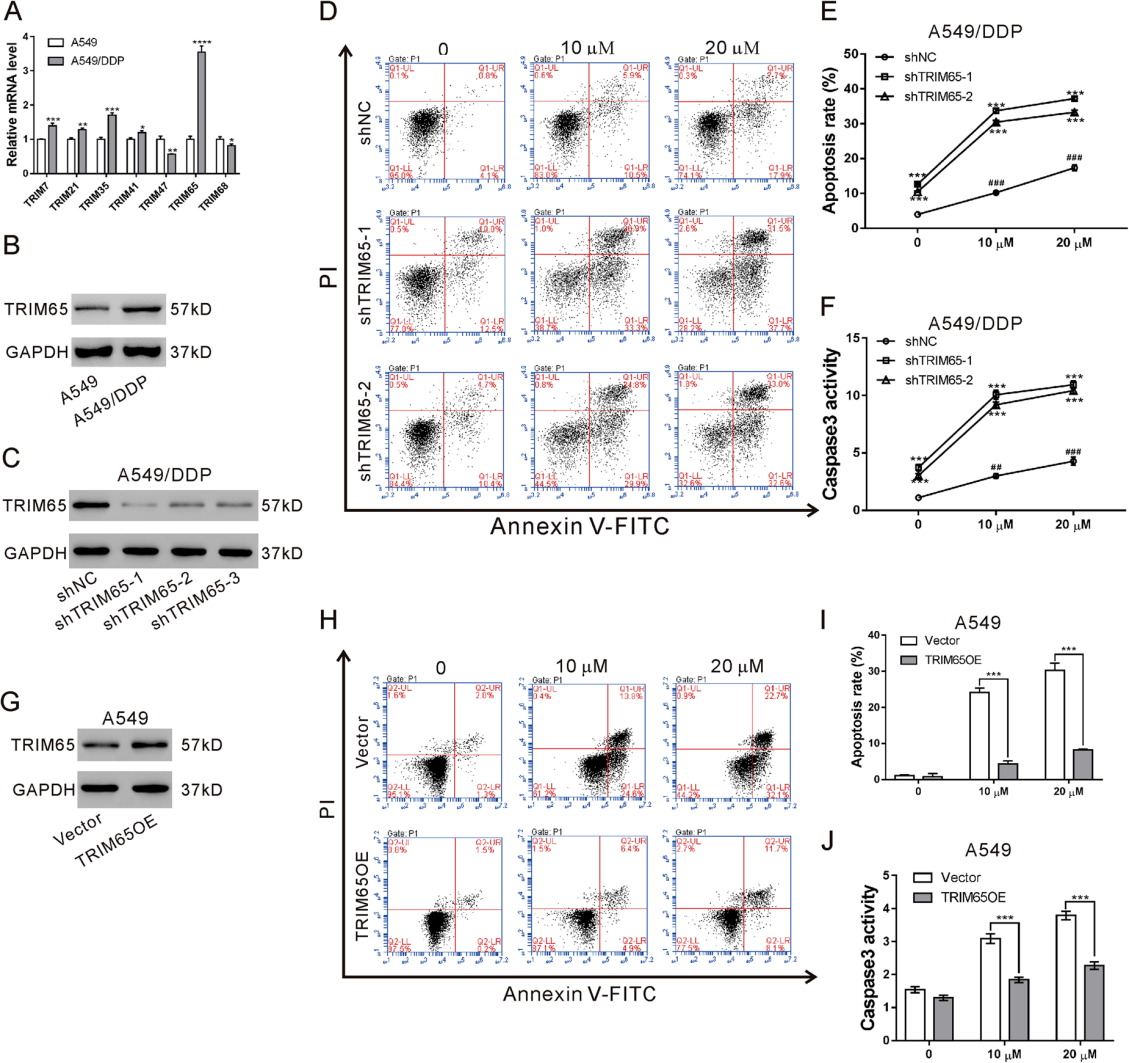
TRIM65 expression was upregulated in A549/DDP cells and involved in cisplatin-induced apoptosis. **a** mRNA expression of TRIM family proteins in A549/DDP and A549 cells. *P < 0.05, **P < 0.01, ***P < 0.001, ****P < 0.0001 versus A549 cells. **b** Protein expression of TRIM65 in A549/DDP and A549 cells. **c** Protein expression of TRIM65 in A549/DDP cells transduced with TRIM65 shRNAs (shTRIM65–1, shTRIM65–2, and shTRIM65–3) or control shRNA (shNC). **d–f** A549/DDP cells transduced with shTRIM65–1, shTRIM65–2 or shNC for 24 h, and then treated with 0, 10, and 20 μM cisplatin for 24 h. Annexin V-PI staining **d, e** and caspase3 activity test **f** were performed to assess early apoptosis. ***P < 0.001 versus shNC and treated with the same concentration of cisplatin; ^###^*P* < 0.001 versus shNC + 0 μM cisplatin. **g** Protein expression of TRIM65 in A549 cells transduced with TRIM65 overexpressing (TRIM65 OE) or Vector virus. **h–j** A549 cells transduced with TRIM65 OE or Vector for 24 h, and then treated with 0, 10, and 20 μM cisplatin for 24 h. Annexin V-PI staining (**d, e**) and caspase3 activity test (**f**) were performed to assess early apoptosis. ***P < 0.001 versus Vector and treated with the same concentration of cisplatin; ^###^*P* < 0.001 versus Vector + 0 μM cisplatin

To investigate the relationship between several TRIM family proteins and the cisplatin resistance in NSCLC, the mRNA expression of several TRIM family proteins was determined in A549 and A549/DDP cells. All the detected TRIM family proteins were more highly expressed in A549/DDP cells than in A549 cells (Fig. 1a), and TRIM65 showed the most significant rise at the transcriptional level (*P* < 0.0001). Western blotting results showed that TRIM65 protein level was also upregulated in A549/DDP cells as compared to that in A549 cells (Fig. 1b).

To explore the biological functions of TRIM65 in cisplatin-resistant human lung cancer cells, TRIM65 expression was knocked down in A549/DDP cells by lentivirus-mediated RNA interference. As shown in Fig. 1c, TRIM65 shRNA (shTRIM65–1, shTRIM65–2, and shTRIM65–3) transduction obviously decreased TRIM65 expression as compared to control shRNA (shNC). shTRIM65–1 and shTRIM65–2 were chosen for the following experiments. A549/DDP cells transduced with shTRIM65–1, shTRIM65–2 or shNC were exposed to DDP, and cell apoptosis was assessed by Annexin V/PI staining (Fig. 1d, e) and caspase3 activity test (Fig. 1f). Cisplatin at 10 and 20 μM significantly induced early apoptosis, and combined treatment with TRIM65 shRNA and cisplatin had a greater effect than cisplatin. It is noticeable that TRIM65 knockdown significantly increased the cell apoptotic rate in A549/DDP cells without cisplatin treatment.

In A549 cells, cisplatin at 10 and 20 μM significantly induced early apoptosis, while TRIM65 overexpression significantly inducingcisplatin resistance in A549 cells (Fig. 1g–j).

### TRIM65 knockdown inhibited autophagy of A549/DDP cells via miR-138-5p/ATG7

**Fig. 2:**
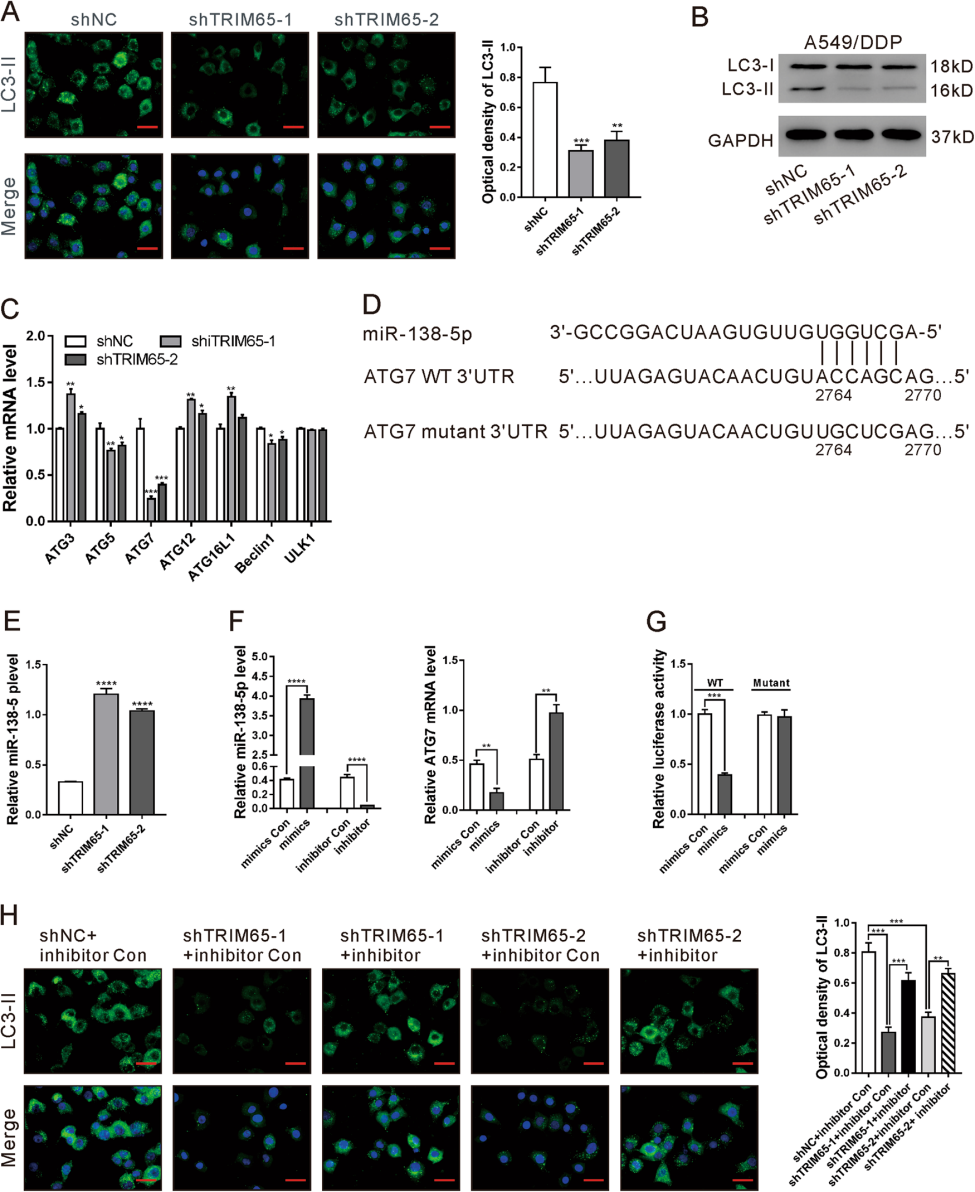
TRIM65 knockdown inhibited autophagy via an miRNA signaling pathway. **a** Immunofluorescence staining of LC3-II in A549/DDP cells transduced with shTRIM65–1, shTRIM65–2, or shNC. Scale bar: 20 μm. **P < 0.01, ***P < 0.001 versus shNC. **b** Western blotting analysis of LC3-II/LC3-I in A549/DDP cells transduced with shTRIM65–1, shTRIM65–2 or shNC. (**c**) mRNA expression of autophagy-related proteins in A549/DDP cells transduced with shTRIM65–1 or shNC. *P < 0.05, **P < 0.01, ***P < 0.001 versus shNC. **d** The potential target sites of miR-138-5p in 3′UTR of the ATG7 gene. **e** The levels of miR-138–5p in A549/DDP cells transduced with shTRIM65–1, shTRIM65–2 or shNC. ****P < 0.0001 versus Vector. **f** A549/DDP cells were treated with miR-138–5p mimics, mimics control (Con), miR-138-5p inhibitor or inhibitor Con for 48 h. The levels of miR-138-5p (left) and ATG7 mRNA (right) were determined by real-time PCR. **P < 0.01, ***P < 0.001. **g** Luciferase reporter assays were performed in A549/DDP cells transfected with miR-138-5p mimics/mimics control and ATG7 3′UTR. WT and Mutant indicate the wild-type and mutated sequences of ATG7 3′UTR, respectively. ***P < 0.001. **h** A549 cells treated with Vector + mimics Con, TRIM65OE + mimics Con, or TRIM65OE + mimics for 48 h. Immunofluorescence staining of LC3-II was performed. Scale bar: 20 μm. **P < 0.01, ***P < 0.001

Autophagy, a highly regulated process, has been linked to chemotherapy resistance^9,10^. Thus, we detected LC3-II, indicative of autophagosomes^32^ in A549 and A549/DDP cells by immunofluorescence staining. A549/DDP cells exhibited more punctate cytosolic staining of LC3-II than A549 cells (Fig. S1A). In addition, TRIM65 knockdown in A549/DDP cells markedly reduced the cytosolic staining of LC3-II (Fig. 2a) and LC3-II/I ratio (Fig. 2b). On the contrary, TRIM65 overexpression elevated LC3-II/I ratio in A549 cells (Fig. S2). These data indicate the role of TRIM65 in the autophagy of A549/DDP cells.

The mRNA expression of autophagy-related proteins was then assessed. TRIM65 knockdown significantly suppressed the mRNA levels of ATG5, ATG7, and Beclin1, of which ATG7 changed the most (*P* < 0.001, Fig. 2c).

TRIM65 is a negative regulator of miRNAs^31^, which are important mediators for drug resistance and autophagy. Previous studies have shown that the expression of miR-224, miR-886, miR-138-5p, miR-27b-3p, miR-194, and miR-100-5p was significantly downregulated in A549/DDP cells in comparison to A549 cells^17,33^. To determine which miRNAs are involved in this process, we tried to find out the miRNA target ATG7 by searching TargetScan (http://www.targetscan.org/), TargetMiner (http://www.isical.ac.in/~bioinfo_miu/target-miner20.htm) and miRDB (http://mirdb.org/miRDB/) databases. We found that, among the above-mentioned six miRNAs, only miR-138-5p may target 3′UTR of the ATG7 gene (Fig. 2d).

Further, we confirmed the decreased expression of miR-138-5p (Fig. S1B) and increased expression of ATG7 (Fig. S1C) in A549/DDP cells as compared with A549 cells. TRIM65 knockdown significantly enhanced miR-138–5p expression in A549/DDP cells (Fig. 2e). miR-138–5p mimics remarkably reduced the mRNA expression of ATG7 as compared to the control (mimics Con), while miR-138-5p inhibitor showed the reverse effect (Fig. 2f). To test whether miR-138-5p could directly target the ATG7 3′UTR in A549/DDP cells, the Luciferase Reporter assay was performed. We observed that miR-138-5p mimics markedly reduced the relative luciferase activity of ATG7 3’′UTR (WT). With a mutated plasmid (Mutant), no significant difference was observed in luciferase activity between the mimics control and miR-138-5p mimics (Fig. 2g). These data suggest that miR-138-5p suppresses the transcription of STG7 by directly binding to the 3′UTR of the ATG7 gene.

To test whether miR-138-5p involves in TRIM65-mediated autophagy, we treated A549/DDP cells with shTRIM65–1/shTRIM65–2 and miR-138-5p inhibitor. As shown in Fig. 2g, miR-138-5p inhibitor obviously abolished the effects of TRIM65 knockdown on LC3-II expression.

### Knockdown of TRIM65 inhibited cisplatin resistance via miR-138-5p

**Fig. 3:**
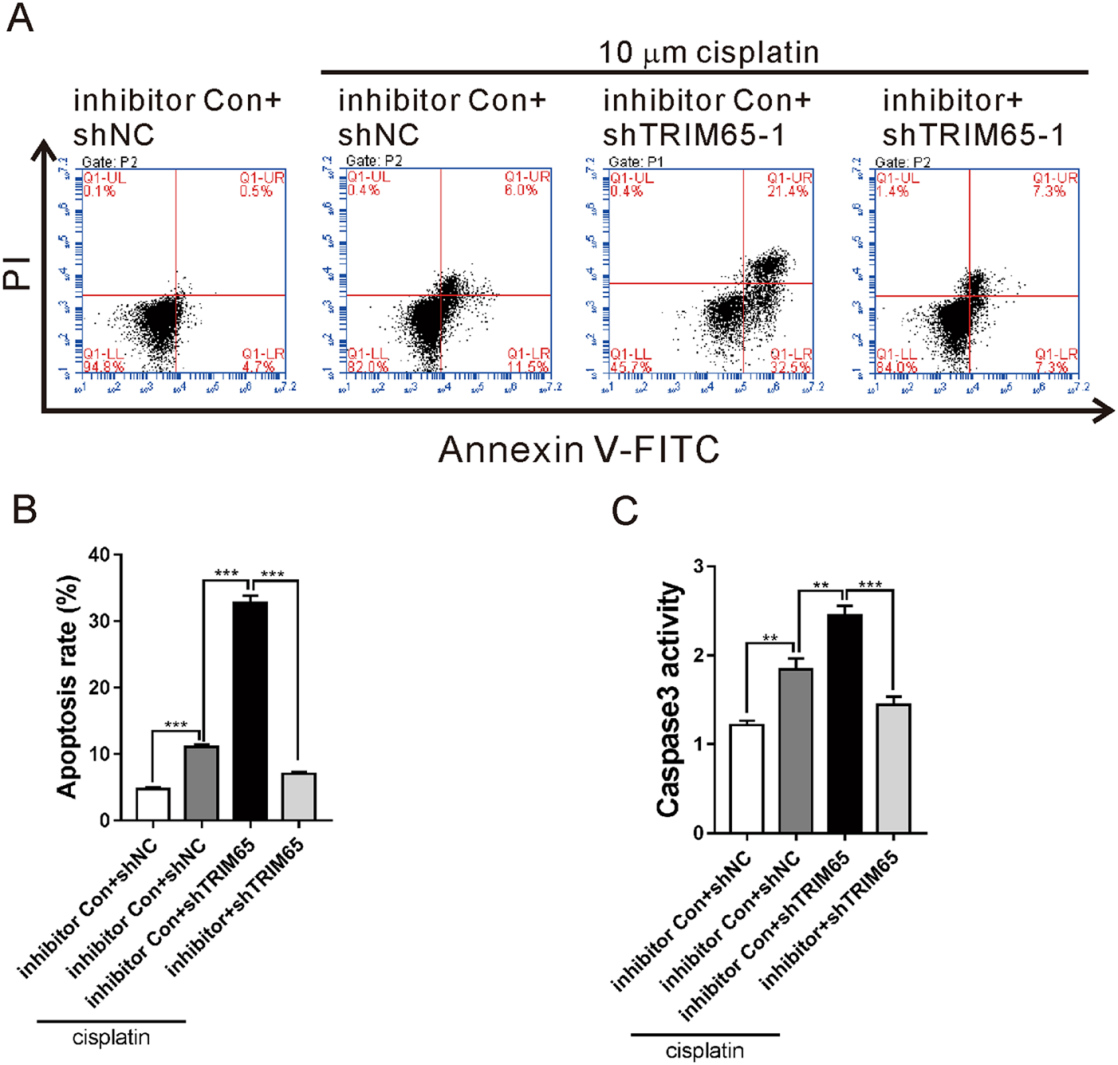
Knockdown of TRIM65 inhibited cisplatin-resistance via miR-138-5p. A549/DDP cells were treated with miR-138-5p inhibitor/inhibitor control (Con) and TRIM65 shRNA (shTRIM65–1)/control shRNA (shNC), with or without 10 μM cisplatin for 48 h. Annexin V-PI staining (**a, b**) and caspase3 activity test (**c**) were performed to assess cell apoptosis. ***P < 0.001

To explore whether miR-138-5p mediated the function of TRIM65 on cisplatin resistance, cell apoptosis was determined in A549/DDP cells treated with shTRIM65–1 and miR-138-5p inhibitor in the presence of 10 μM cisplatin. As illustrated in Fig. 3, shTRIM65–1 caused a significant increased apoptosis in A549/DDP cells exposed to cisplatin, while miR-138-5p inhibitor significantly abolished the effects of TRIM65 knockdown. These data indicate that miR-138-5p is involved in TRIM65-mediated cisplatin resistance.

### TRIM65 induced the ubiquitination and degradation of TNRC6A in A549 cells

**Fig. 4:**
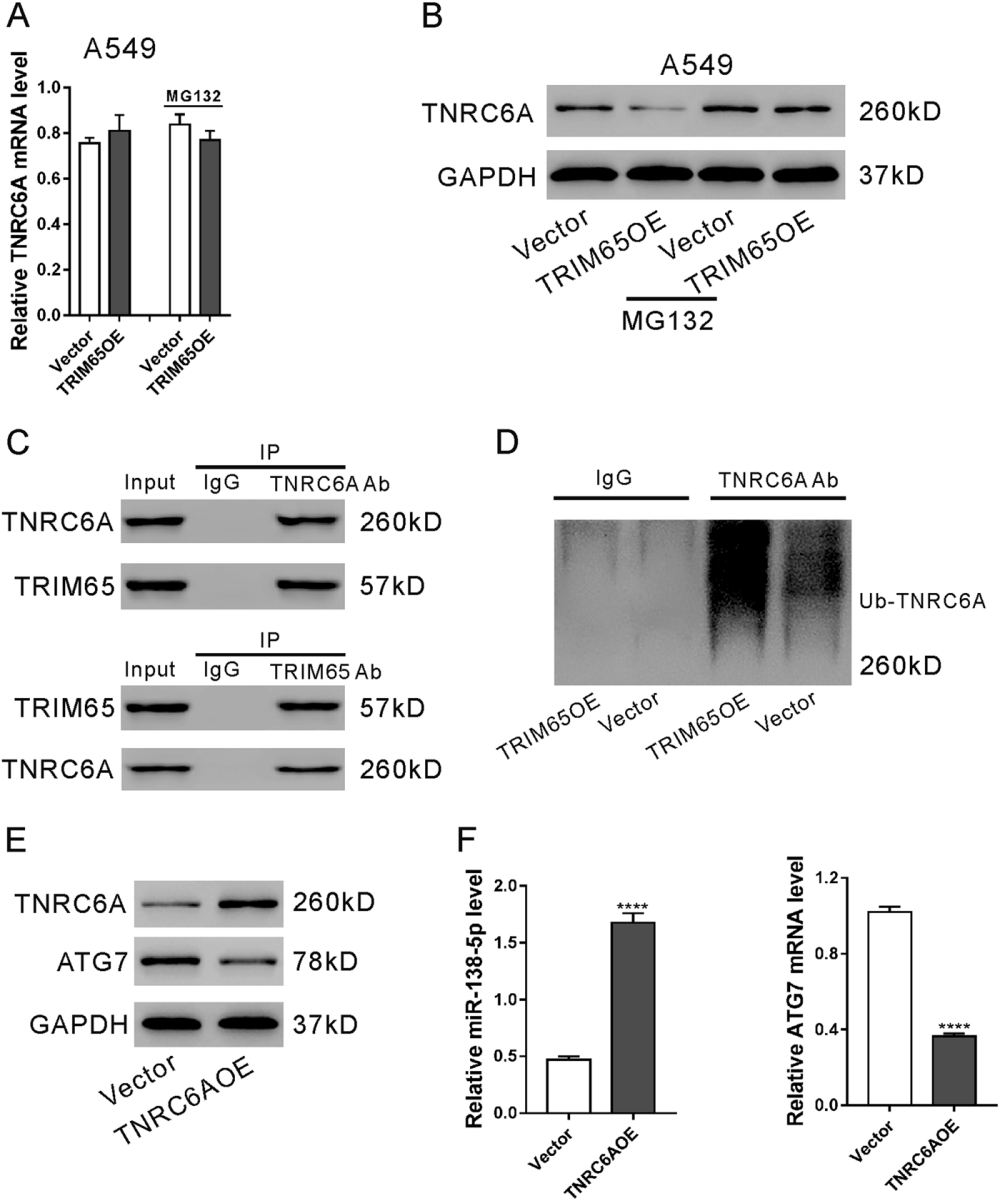
TRIM65 regulated the ubiquitination and degradation of TNRC6A in A549 cells. **a, b** A549 cells were transduced with TRIM65 overexpressing lentivirus (TRIM65OE) or Vector lentivirus in the presence and absence of 10 μM MG132 for 48 h. The mRNA (**a**) and protein (**b**) expression of TNRC6A was assessed. **c** Cell lysates from A549 cells were immunoprecipitated (IP) with TNRC6A antibody (Ab) or TRIM65 Ab or IgG, and then subjected to western blotting analysis with TNRC6A Ab or TRIM65 Ab. The lanes labeled Input indicate cell lysate. **d** A549 cells transduced with TRIM65OE or Vector were lysed, IP with TNRC6A Ab and then subjected to western blotting analysis with ubiquitin Ab. **e, f** A549 cells were transduced with TNRC6A overexpressing lentivirus (TNRC6A) or Vector for 48 h. Western blotting (**e**) and real-time PCR analyses (**f**) were done. ****P < 0.0001 versus Vector

TRIM65 is known to negatively regulate miRNAs by ubiquitinating TNRC6A^31^. Here, we found that TRIM65 overexpression had no effects on the mRNA expression of TNRC6A (Fig. 4a), but obviously reduced the protein expression of TNRC6A (Fig. 4b). Additional treatment of MG132, a proteasome inhibitor, abolished the effects of TRIM65 overexpression on TNRC6A protein, which indicated that TRIM65 downregulation of TNRC6A in A549 cells was mediated by a proteasome-related pathway. Immunoprecipitation experiments showed that TRIM65 bound with TNRC6A in A549 cells (Fig. 4c), and that TRIM65 overexpression induced the ubiquitination of TNRC6A (Fig. 4d). These data suggest that TRIM65 downregulates TNRC6A through a post-translational modification in A549 cells.

As expected, ectopic expression TNRC6A significantly increased miR-138-5p and decreased the target gene of miR-138-5p, ATG7 (Fig. 4e, f).

### Knockdown of TRIM65 inhibited cisplatin-resistant tumor growth in vivo

**Fig. 5:**
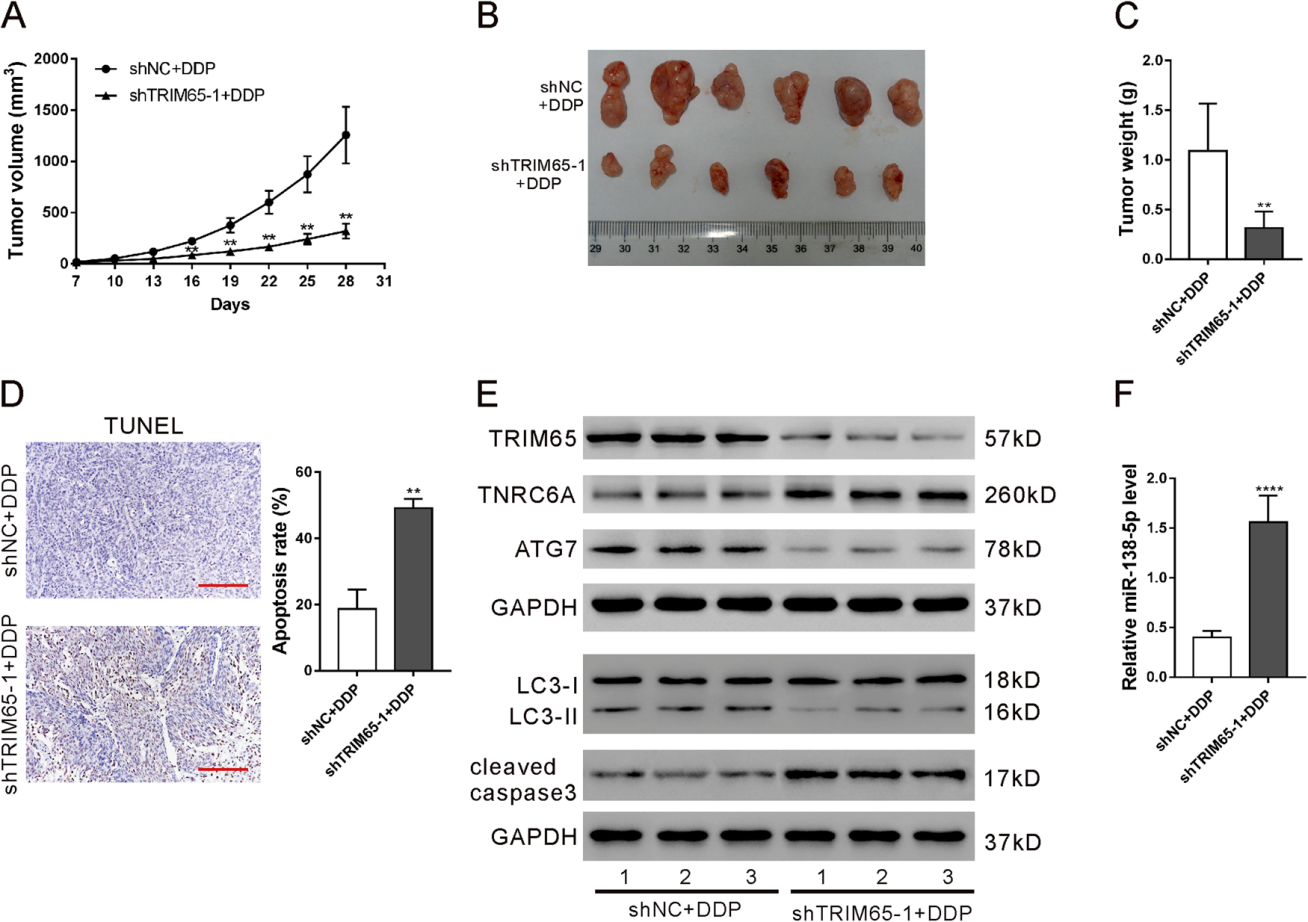
Knockdown of TRIM65 inhibited cisplatin-resistant tumor growth in vivo. BALB/c nude mice were injected with A549/DDP cells (5 × 10^6^) transduced with shTRIM65–1 and shNC, respectively (*n* = 6 per group). After 1 week (Day 7), the mice were intraperitoneally administered with cisplatin (5 mg/kg) every week for 3 weeks. **a** Tumor volume was estimated every 3 days. **b–f** On Day 28, the xenografts were resected (**b**) and weighed (**c**). TUNEL (**d**), western blotting (**e**) and real-time PCR (**f**) analyses were done to assess cell apoptosis rate, protein expression and miR-138-5p level, respectively. **P < 0.01, ****P < 0.0001

To confirm the function of TRIM65 on cisplatin resistance in vivo, we established A549/DDP cell lines stably expressing TRIM65 shRNA (shTRIM65–1) or control shRNA (shNC), and these cells were injected subcutaneously to construct a mouse xenograft model. One week after subcutaneous implantation (Day 7), the mice were treated with cisplatin for 3 weeks. Slower tumor growth rates were detected in the shTRIM65–1 group as compared to the shNC group at the interval between Day 16 and Day 28 (Fig. 5a). On Day 28, the xenografts were recovered. The tumor size (Fig. 5b) and tumor weight (Fig. 5c) were also notably reduced, but cell apoptosis was remarkably increased in the shTRIM65–1 group in comparison to the shNC group. The shTRIM65–1 group displayed downregulated levels of TRIM65, ATG7, and LC3-II/LC3-I (Fig. 5e), and upregulated levels of TNRC6A, cleaved caspase3, (Fig. 5e) and miR-138-5p (Fig. 5f). These results indicate that TRIM65 knockdown inhibited the growth of tumors derived from A549/DDP cells.

### Expression of TRIM65 and miR-138-5p in the cisplatin non-resistant and resistant NSCLC tissues

**Fig. 6:**
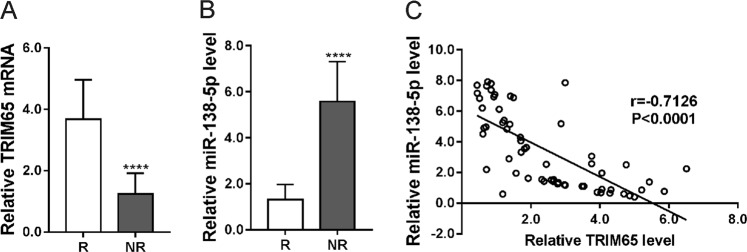
Expression of TRIM65 and miR-138-5p in the cisplatin non-resistant (NR) and resistant (R) NSCLC tissues. Real-time PCR analysis of TRIM65 (**a**) and miR-138-5p (**b**) was performed in NR and R tissues (*n* = 30), and then correlation analysis between TRIM65 and miR-138-5p expression was carried out. ****P < 0.0001

Further, we analyzed TRIM65 and miR-138-5p expression in the cisplatin non-resistant and resistant NSCLC tissues. Cisplatin-resistant NSCLC tissues displayed higher expression of TRIM65 mRNA (Fig. 6a) and lower expression of miR-138-5p (Fig. 6b) as compared to that in cisplatin non-resistant ones. A negative correlation was observed between miR-138-5p expression and TRIM65 mRNA in NSCLC tissues (Fig. 6c). These data clinically suggest the involvement of TRIM65 and miR-138-5p in cisplatin resistance.

## Discussion

Evidence has shown that TRIM proteins promote chemoresistance of various human cancers, such as TRIM8 in colorectal cancer^26^, TRIM32 in breast cancer^28^, TRIM14 in gliomas^29^, and TRIM31 in pancreatic cancer^30^. TRIM65 is overexpressed in lung cancer tissues and may act as an oncogene in lung cancer by promoting lung cancer proliferation, migration, and invasion^34^. It is unclear whether TRIM65 is involved in chemotherapy resistance. Cisplatin resistance is the main cause of the treatment failure in patients with NSCLC. In the current study, we found the upregulation of TRIM65 in cisplatin-resistant NSCLC tissues as compared to that in non-resistant ones. TRIM65 knockdown suppressed NSCLC chemoresistance in vitro as well as in vivo after cisplatin treatment, suggesting targeting TRIM65 may be a promising strategy to enhance chemotherapy response for NSCLC.

Autophagy is a highly regulated process that can disassemble unnecessary or dysfunctional components. In cancer, autophagy plays crucial roles in adaptive responses to stress, which promotes cell survival^35^. Autophagy is identified as a mechanism of resistance to chemotherapy^9,10^. Consistently with the previous findings^36^, a higher autophagy activity was observed in A549/DDP cells than in A549 cells (Fig S1). Autophagy-specific markers LC3-II and ATG7 were reduced in A549/DDP cells with TRIM65 knockdown, which indicated the role of TRIM65 in autophagy. Apoptosis, the best-described form of programmed cell death, is commonly activated during chemotherapy. Here, tumors derived from cells expressing TRIM65 shRNA had decreased growth rate, reduced levels of ATG7 and LC3-II/I, and increased cleaved caspase3 and apoptotic cell ratio after cisplatin treatment. TRIM65 may function in chemoresistance by connecting the autophagy and apoptosis process, although the precise mechanism is to be investigated.

TRIM65 is identified as an E3 ligase for TNRC6A, which is a component of RNA-induced silencing complex (RISC) and participates in miRNA-induced gene silencing^31^. In line with the previous findings, our immunoprecipitation experiments performed in A549 cells demonstrated that TRRIM65 interacted with TNRC6A and promoted the ubiquitination of the latter protein. miRNAs are important mediators for drug resistance and autophagy. Thus, we explored putative miRNAs targeting ATG7 and involving in cisplatin-resistance. By searching previous literatures^17,33^ and web-accessible miRNA target databases, we found that miR-138-5p was the most likely miRNA. Further experiments showed that miR-138-5p directly bound to the 3′UTR of ATG7, which reduced ATG7 mRNA expression. The participation of miR-138-5p in chemosensitivity has been studied in NSCLC cells^37^ and leukemia cells^38^. Here, cisplatin-resistant NSCLC cell lines and tissues had lower expression of miR-138–5p than cisplatin non-resistant controls, suggesting the involvement of miR-138–5p in cisplatin resistance. In a previous study on pancreatic cancer, miR-138-5p is shown to suppress serum starvation-induced autophagy by targeting SIRT1, but not by ATG7^39^. The inconsistency between the above study and ours may be ascribed to the different cell types. Moreover, miR-138-5p expression was negatively correlated with TRIM65 mRNA in NSCLC tissues. TRIM65 knockdown significantly increased miR-138-5p, and miR-138-5p inhibitor significantly abolished the effects of TRIM65 knockdown on autophagy and cisplatin-induced apoptosis. Collectively, these data suggest that miR-138-5p mediates the function of TRIM65 on autophagy and cisplatin resistance.

In summary, by studying cell lines, mouse models, and clinical samples, we have identified the novel function of TRIM65 on autophagy and cisplatin resistance in NSCLC through a miRNA-mediated pathway. TRIM65 may be used as a potential therapeutic target for the treatment of cisplatin-resistant NSCLC.

## Materials and methods

### Cell culture

The human NSCLC cell line A549 and the cisplatin-resistant A549 cell line (A549/DDP) (obtained from JRDUN Biotech., Shanghai, China) were cultured in RPMI 1640 medium (Hyclone, Logan, UT, USA) supplemented with 10% fetal bovine serum (FBS; Gibco, Carlsbad, CA, USA). All cultures were maintained in a humidified atmosphere containing 5% CO2 at 37 °C.

### RNA interference and gene overexpression

Oligonucleotides encoding TRIM65 short hairpin RNAs were ligated at the AgeI/EcoRI sites of pLKO.1 (Addgene, Cambridge, MA, USA). The target sites were as follows: shTRIM65–1, GCTACAGGCCCTGGAAATA; shTRIM65–2, GACCTGAAGCAGTTGCTAA; shTRIM65–3, GAGGAAACTCTGGCAGAAT.

The cDNA sequence of human TRIM65 and TNRC6A gene was amplified using the following primers: TRIM65 forward primer, 5′–CGGAATTCATGGCCGCGCAGCTG-3′ and reverse primer, 5′-CGGGATCCTCAGCTGAGCACCTCTTCC-3′; TNRC6A forward primer, 5′-CGGAATTCATGAGAGAATTGGAAGCTAAAGC-3′ and reverse primer, 5′- CGGGATCCTTACATGGACTCTCCACCC-3′. After digestion, the fragments were ligated into the pLVX-puro vector (Clontech, Palo Alto, CA, USA) between the EcoRI and BamHI sites to make the TRIM65- and TNRC6A-overexpressing construct, respectively.

To prepare lentivirus, the constructs expressing TRIM65 shRNAs, scramble shRNA (shNC), TNRC6A or vector were co-transfected into 293 cells with packaging plasmids using Lipofectamine 2000 (Invitrogen, Carlsbad, CA, USA) as previously described^40^. At 48–72 post transfection, lentiviruses were collected from the culture medium and used to transduce A549/DDP cells.

### Transfection of mimics and inhibitor

miR-138-5p mimic, inhibitor (50 nM), or negative control were transfected into cells with Lipofectamine 2000 (Invitrogen) as the manufacturer’s instructions suggested. The mimic and inhibitor sequences were as follows: miR-138-5p mimics: 5′-AGCUGGUGUUGUGAAUCAGGCCG-3′; mimics control (Con): 5′-CAGUACUUUUGUGUAGUACAA-3′; miR-138-5p inhibitor: 5′-CGGCCUGATTCACAACACCAGCT-3′.

### Real-time PCR analysis

Total RNA was isolated using TRIzol Reagent (Invitrogen) as per the manufacturer’s instructions. To analyze mRNA levels of target genes, total RNA was reverse transcribed into cDNA with random primers using MMLV reverse transcriptase (Promega, Madison, WI, USA) according to the manufacturer’s instructions. To quantify miR-138-5p level, total RNA was reverse transcribed with the specific reverse transcription (RT) primer. Real-time PCR was carried out in triplicate on an ABI 7500 instrument (Applied Biosystems, Foster City, CA, USA). GAPDH and U6 small nuclear RNA (snRNA) were amplified as an internal control for mRNA and miRNA expression, respectively. All the primers are listed in Table 1.

**Table 1:** Oligonucleotide primers for real-time PCR

Gene	Primers
TRIM35	Forward: 5′ CCCACCAGGAAATGAGATAG 3′ Reverse: 5′ GAAGTCAGCAGAGACAAGAG 3
TRIM68	Forward: 5′ CAGAAACTGCCAGACAATCC 3′ Reverse: 5′ AGCCTTATCACCCAGAATCC 3′
TRIM47	Forward: 5′ CGCAGCTTCTCCGTCTGGTTTC 3′ Reverse: 5′ CCAAGGCACGGTCAGCGTATTC 3′
TRIM7	Forward: 5′ AGGGTGTCCACATAATTGTTG 3′ Reverse: 5′ ATGCCATGAGGCTCTTTATTG 3′
TRIM21	Forward: 5′ CCCTTTGCTGGGTATGTG 3′ Reverse: 5′ TGCTCCCTCTCATCCTTC 3′
TRIM41	Forward: 5′ TTTGGGCAAGGCAACATC 3′ Reverse: 5′ TGGTGCGTGGTTTCAATC 3′
TRIM65	Forward: 5′ CTCAACCCTGTCACTAAGC 3′ Reverse: 5′ CACCTCCCAGAACAAGAAG 3′
ATG3	Forward: 5′ TGTTTGGCTATGATGAGCAACG 3′ Reverse: 5′ CACATGGGAGGTGGTGGC 3′
ATG5	Forward: 5′ GATCACAAGCAACTCTGGATGG 3′ Reverse: 5′ AGCCACAGGACGAAACAGC 3′
ATG7	Forward: 5′ CCAGTGACGCCAGATTTCC 3′ Reverse: 5′ GGCAGGCACAGATGCTATG 3′
ATG12	Forward: 5′ AGAGCGAACACGAACCATCC 3′ Reverse: 5′ CCCACGCCTGAGACTTGC 3′
ATG16L1	Forward: 5′ TGACCTGGAGACGGAGTGC 3′ Reverse: 5′ ACTGGTAGAGGTTCCTTTGCTG 3′
Beclin1	Forward: 5′ AAACCAGATGCGTTATGCCC 3′ Reverse: 5′ TTTCCGTAAGGAACAAGTCGG 3′
ULK1	Forward: 5′ CAAGAAGAACCTCGCCAAGTC 3′ Reverse: 5′ GGAAGAGCCTGATGGTGTCC 3′
ATG16L1	Forward: 5′ TGACCTGGAGACGGAGTGC 3′ Reverse: 5′ ACTGGTAGAGGTTCCTTTGCTG 3′
TNRC6A	Forward: 5′ CTGAGTTTGCCAGTGAAGAG 3′ Reverse: 5′ GCACCATTCCAGTGATTGAG 3′
GAPDH	Forward: 5′ AATCCCATCACCATCTTC 3′ Reverse: 5′ AGGCTGTTGTCATACTTC 3′
miR-138-5p	RT primers: 5′ GTCGTATCCAGTGCAGGGTCCGAGGTATTCGCACTGGATA CGACCGGCCT 3’ Forward: 5′ GCGAGCTGGTGTTGTGAATC 3′ Reverse: 5′ AGTGCAGGGTCCGAGGTATT 3′
RNU6–1	Forward: 5′ CTCGCTTCGGCAGCACA 3′ Reverse: 5′ AACGCTTCACGAATTTGCGT 3′

### Western blot analysis

After washing twice with ice-cold phosphate-buffered saline (PBS), the cells were incubated at 4 °C with RIPA buffer supplemented with a proteinase inhibitor cocktail (JRDUN biotech, Shanghai, China) for 30 min. Subsequently, the samples were centrifuged at 12,000 r.p.m. for 15 min at 4 °C, and the supernatant was collected for sodium dodecyl sulfate-polyacrylamide gel electrophoresis (SDS-PAGE). Protein on the gels were electrophoretically transferred to a nitrocellulose membrane (Millipore, Bredford, MA, USA), and probed with primary antibodies against TRIM65 (Abcam, Cambridge, MA, USA; 1:1000 dilution), TNRC6A (Abcam; 1:1000 dilution), ATG7 (Abcam; 1:200 dilution), LC3 (Abcam; 1:2000 dilution) or cleaved caspase3 (Abcam; 1:1000 dilution). After incubation with an HRP-conjugated secondary antibody (Beyotime, Shanghai, China; 1:1000 dilution), signals were detected using an enhanced chemiluminescence (ECL) detection kit (Millipore). The membrane was probed with a primary antibody against GAPDH (Cell Signaling Technology, Danvers, MA, USA; 1:2000 dilution) for equal loading control.

### Cell apoptosis analysis

The percentages of cells undergoing apoptosis were measured by Annexin V-fluorescein isothiocyanate (FITC) apoptosis detection kit (Beyotime). A549/DDP cells were plated in six-well plates (3.0 × 10^5^ per well) and treated as indicated. Subsequently, the cells were collected, washed once with PBS, incubated with Annexin V and propidium iodide (PI) following the manufacturer’s protocol, and analyzed by a flow cytometer (BD Biosciences, Franklin Lakes, NJ, USA).

### Caspase3 activity test

The activity of Caspase3 was measured by a colorimetric assay kit (KeyGEN Biotech, Nanjing, China) as per the manufacturer’s protocol.

### Fluorescence microscopy

Cells grown on cover slips were treated as indicated and washed twice with PBS. Following fixation with 4% paraformaldehyde and permeabilization with 0.1% Triton X-100, the cells were washed and blocked with 1% BSA for 30 min. Subsequently, cells were probed with the primary rabbit antibody against LC3-II (Abcam; 1:1000 dilution) overnight at 4 °C, followed by incubation with Alexa Fluor 488-conjugated goat anti-rabbit secondary antibody (Beyotime, 1:500 dilution) for 60 min. The nuclei were stained with 4′,6-diamidino-2-phenylindole dihydrochloride (DAPI) (Beyotime) for 10 min. The samples were observed on a fluorescence microscope (Leica microsystems, Deerfield, IL, USA).

### Luciferase assays

The wild type (WT, containing putative miR-138-5p binding sites) or mutate (mutant, putative binding sites were mutated) 3′UTR of ATG7 were constructed into the pGL3-Enhancer Vector (Promega). The constructs were co-transfected with pRL-TK (Promega), and miR-138-5p mimics or mimics control (Con). The pRL-TK plasmid was used as a transfection control. Luciferase activity was measured using a luciferase assay kit (Promega, Madison, WI, USA) at 48 h after transfection.

### Immunoprecipitation (IP)

Cell lysate was prepared with RIPA buffer as mentioned above, incubated with antibody against TRIM65 (Abcam), TNRC6A (Abcam) or IgG (Santa Cruz Biotech., Santa Cruz, CA, USA) for 2 h, and then with protein A/G Plus agarose beads (Santa Cruz Biothech.) for 1 h. Immunoprecipitates were washed three times with RIPA buffer, fractionated by SDS-PAGE, and detected by western blotting with primary antibodies against TRIM65, TNRC6A, and ubiquitin (Abcam).

### Xenograft experiments

To evaluate the in vivo effects of TRIM65, BALB/c athymic nude mice (4–5 week old) were randomly divided into two groups (*n* = 6) and injected with 5 × 10^6^ A549/DDP cells stably transduced with shTRIM65–1 and shNC, respectively. After 1 week (Day 7), the mice were intraperitoneally administered with cisplatin (5 mg/kg, Chinese medicine reagent, Beijing, China) every week for 3 weeks. Tumor volume was estimated every 3 days. On Day 28, mice were euthanized, and the xenografts were collected and weighed. The xenografts were subjected to real-time PCR, western blotting, and TUNEL (Terminal deoxynucleotidyl transferase dUTP Nick-End Labeling) analyses (Roche, Indianapolis, IN, USA). All animal experiments were approved by the Committee on Animal Care and Use of Shanghai Chest Hospital.

### Human lung cancer specimen collection

A total of 60 NSCLC subjects (30 cisplatin non-resistant and 30 cisplatin resistant) were collected at the Department of Thoracic Surgery, Shanghai Chest Hospital with written consent from the patients. The study was approved by the Institute Research Ethics Committee of Shanghai Chest Hospital.

### Statistical analysis

Each experiment was repeated independently at least three times. Graphpad Prism Software (Graphpad Prism, San Diego, CA, USA) was used for the statistical analyses. Two-tailed Student’s *t*-test was performed for statistical significance analysis between two groups, and one-way analysis of variance (ANOVA) was used for more than two groups. Pearson correlation analysis was done to identify the correlation between TRIM65 mRNA and miR-138-5p in NSCLC tissues. *P* < 0.05 was considered statistically significant.

## Supplementary information

Figure S1-S2
