# Supplementary material for: Knockdown of TRIM65 inhibits autophagy and cisplatin resistance in A549/DDP cells by regulating miR-138-5p/ATG7
Source: Cell Death Dis. 2019 Jun 3;10(6):429. doi: 10.1038/s41419-019-1660-8 (PMC6546683; doi:10.1038/s41419-019-1660-8)
Supplement: Supplementary file 1 — Figure S1-S2 [file 41419_2019_1660_MOESM1_ESM.docx]

**Knockdown of TRIM65 inhibits autophagy and cisplatin resistance in A549/DDP cells through regulating miR-138-5p/ATG7**

**Running title:** TRIM65 and cisplatin resistance

Xufeng Pan^*^, Yong Chen^*^, Yuzhou Shen, Jicheng Tantai^#^

Department of Thoracic Surgery, Shanghai Chest Hospital, Shanghai Jiao Tong University, Shanghai, China

^#^Corresponding to: Dr. Jicheng Tantai, Address: No.241 West Huaihai Road, Shanghai 200030, China; Tel: 86-21-62821990; Email: jcttai@163.com.


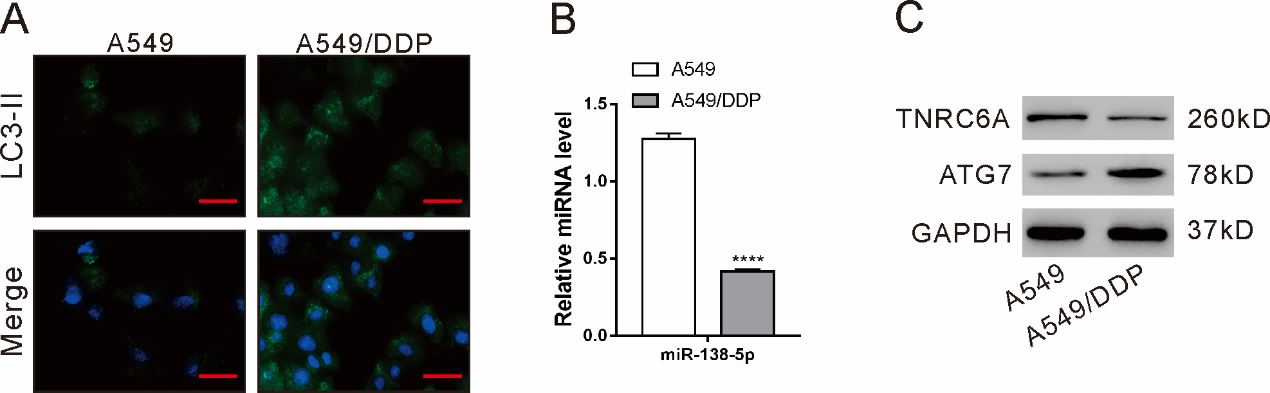


**Figure S1.** Immunofluorescence staining of LC3-II (A) in A549 and A549/DDP cells. Scale bar: 20 μm. Expression of miR-138-5p (B) and rotein levels of TNRC6A and ATG7 (C) in A549 and A549/DDP cells. ****P<0.001 versus A549.

**
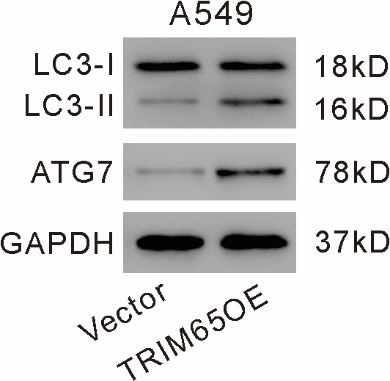
**

**Figure S2.** Western blotting analysis of LC3-II/LC3-I and ATG7 in A549 cells transduced with TRIM65 overexpressing (TRIM65 OE) or Vector virus.
